# Launching *Animal Diseases*: animal warfare and health, food safety, and public health

**DOI:** 10.1186/s44149-021-00006-8

**Published:** 2021-04-23

**Authors:** Zhen F. Fu, Huanchun Chen

**Affiliations:** 1 Department of Pathology, College of Veterinary Medicine, University of Georgia, Athens, GA 30602 USA; 2 State Key Laboratory of Agricultural Microbiology, College of Veterinary Medicine, Huazhong Agricultural University, Wuhan, 430070 Hubei China

It is indeed our great pleasure to have the inaugural issue of the *Animal Diseases*, an open access and fast peer-reviewed journal, published. Animal diseases not only cause enormous economic losses (more than 20%) to the animal farming industry, but also pose serious threats to human health as more than 60% human infectious diseases originate in animals. Therefore, it is absolutely imperative to conduct research on all aspects of animal diseases as well as to develop novel drugs, vaccines, diagnostic methods and technologies to address above challenges.

To promote One Health initiative, *Animal Diseases* is committed to publish high quality manuscripts in animal disease research/application which are closely related to human health, including the latest state-of-the-art articles from academia, government laboratories and industry experts. The journal publishes original research, review, case report and other types of communications in all areas of animal diseases, infectious or non-infectious. It has partnered with BMC, part of Springer-Nature, and a pioneer of open access publishing. Initially electronic publication will proceed as manuscripts are accepted.

## Our inaugural issue

The inaugural issue is a great example of manuscripts *Animal Diseases* aims to publish. The global outbreaks of human coronavirus diseases, including severe acute respiratory syndrome (SARS), Middle East respiratory syndrome (MERS) and the ongoing pandemic COVID-19 have aroused extensive public attention. The origins and transmission pathways for these diseases remain unknown but are universally regarded as originated from animals. *Animal Diseases* provides a new platform for rapid communication of scientific information about coronavirus and other pathogens.

SARS, MERS, and COVID-19 are most likely originated from bats and transmitted to humans through intermediate hosts. In this inaugural issue, researchers from the CAS key laboratory of special pathogens, Wuhan Institute of Virology, summarize the current knowledge of bat SARS-related CoVs on geographical distribution, genetic diversity, the potential for cross-species transmission and possible pathogenesis in humans, aiming for a better understanding, prevention and control of these diseases (Geng and Zhou 2021).

CoVs belong to the *Coronaviridae* family of the *Nidovirales* order. Recently, advanced technologies for viral detection and viral genome analyses have enabled scientists to characterize many new nidoviruses, which has greatly expanded the *Nidovirales* order with new classification and nomenclature. Zhou et al. (2021) provides an overview of the latest research progresses about classification, host range, genetic diversity, genomic pattern and pathogenic features of coronaviruses. The information will promote understanding of the phylogenetic relationship and transmission of pathogenic coronaviruses and will benefit virological research and viral disease control.

Replication is one of the critical processes for virus infection. Bile acids (BAs) are synthesized in the liver from cholesterol to facilitate the absorption of fat-soluble nutrients and act as signaling molecules that modulate various biological functions. BAs present either pro-viral or anti-viral effects for the replication of enteric viruses *in vivo* and *in vitro* (Kong et al. 2021). A review about the role of BAs in virus infections is included in the first issue of *Animal Diseases* (Kong et al. 2021). Current information on biosynthesis, transportation and metabolism of BAs and the influence of BAs on enteric virus replication, such as caliciviruses, rotaviruses, and coronaviruses are presented. The application of BAs for cell culture adaptation of fastidious enteric caliciviruses and control of infection by these viruses are also discussed. This article provides novel insights into the development of antivirals and/or disinfectants for enteric viruses.

The zoonotic cryptosporidiosis is globally distributed, one of the major diarrheal diseases in humans and animals. The enteric *Cryptosporidium* is a globally distributed, water-borne and food-borne diarrheal-causing parasite (Zhu et al. (2021), which makes it one of the major environmental concerns. It may serve as one of the model One Health pathogens that impact humans, animals and environment at regional and global levels. Despite its importance, fully effective drugs are not yet available. Zhu et al. (2021) systematically summarizes the unique characteristics for the parasite biology and challenges/progresses in developing therapeutics for cryptosporidiosis.

With the vigorous development of new technologies, “omics” tools have been widely used in the research of animal and human diseases. Metabolomics can qualitatively and quantitatively measure small endogenous metabolites in tissues, cells or biological fluids, detect metabolic changes, screen out differential metabolites and further reveal the related metabolic pathways. In recent years, medicinal plants have attracted much attention in the prevention and treatment of osteoporosis without causing side effects (Yu et al. 2021). In the inaugural issue of *Animal Diseases,* we present an exciting study which combines traditional Chinese herb *Epimedium* with modern omics techniques (Yu et al. 2021). A serum metabolomics assay was performed to investigate the use of icariin (ICA), the main bioactive component isolated from *Epimedium*, in low-calcium diet-induced Cage layer osteoporosis (CLO), which known as a bone metabolic disease. One highlight from this study is that ICA can effectively prevent bone loss in low-calcium diet-induced CLO by mediating steroid biosynthesis and glycerophospholipid metabolism.

These studies provide a glimpse about the main scope of research published in *Animal Diseases*. It is hoped that this inaugural issue would provide a fresh perspective on the understanding of animal diseases as well as the inter-relationship between animals and humans. We expected that our journal, *Animal Diseases,* will play a major role in the aspirational challenges of animal warfare and health, food safety, and public health.
